# Pharmacologic Targets and Prototype Therapeutics in the Kallikrein-Kinin System: Bradykinin Receptor Agonists or Antagonists

**DOI:** 10.1100/tsw.2006.226

**Published:** 2006-10-09

**Authors:** J. N. Sharma, G.J. AL-Sherif

**Affiliations:** Department of Applied Therapeutics Health Sciences Centre Kuwait University, Safat, 13110, Kuwait

**Keywords:** bradykinin, bradykinin agonists, bradykinin antagonists, kallikrein-kinin system, inflammation, cardioprotection

## Abstract

The kallikrein-kinin system (KKS) is a complex system produced in various organs. This system includes kininogen (precursor for kinin), kallikreins, and pharmacologically active bradykinin (BK), which is considered to be proinflammatory and/or cardioprotective. It is a proinflammatory polypeptide that is involved in many pathological conditions and can cause pain, inflammation, increased vascular permeability, vasodilation, contraction of various smooth muscles, as well as cell proliferation. On the other hand, it has been shown that BK has cardioprotective effects, as all components of KKS are located in the cardiac muscles. Numerous observations have indicated that decreased activity of this system may lead to cardiovascular diseases, such as hypertension, cardiac failure, and myocardial infarction. BK acts on two receptors, B_1_ and B_2_, which are linked physiologically through their natural stimuli and their common participation in a variety of inflammatory responses. Recently, numerous BK antagonists have been developed in order to treat several diseases that are due to excessive BK formation. Although BK has many beneficial effects, it has been recognized to have some undesirable effects that can be reversed with BK antagonists. In addition, products of this system have multiple interactions with other important metabolic pathways, such as the renin-angiotensin system.

### INTRODUCTION TO THE KALLIKREIN-KININ SYSTEM

The kallikrein-kinin system (KKS) is complex, with several bioactive peptides that are formed in vascular smooth muscle as well as in the heart. The main constituents of this system are enzymes, such as kallikreins, protein precursors that are kininogens, and the potent vasoactive peptide kinin.

Kallikreins are serine proteases found in glandular cell, neutrophils, and biological fluids. They are divided into two groups: tissue kallikrein and plasma kallikrein. Both differ in molecular weight, amino acid composition, type of kinin released, and functions.

Kinins are the vasoactive components in this system that are released through the actions of kallikreins on kininogens. In humans, kinin refers to bradykinin (BK), kallidinin, and carboxyterminal des-Arg metabolites. Also, T-kinins(Ile-Ser-BK) and Met-T-kinin have been found only in rats. Kinins mediate a variety of physiological actions related to cardiovascular homeostasis, inflammatory, algesic responses, and pain-transmitted mechanisms[1]. These actions are promoted by activation of at least two receptor subtypes: $B_1$ and $B_2$ receptors. Also, kinins activate endothelial cells, resulting in vasodilation, increased vascular permeability, production of nitric oxide (NO), and releasing tissue type plasminogen activator (t-PA).

Kinins, BK, and lysyl-BK are important mediators of inflammatory responses. They are liberated from precursor molecule kininogen by various proteases known as kininogenases. There are three types of kininogens: high (HMW) and low (LMW) molecular weight kininogen and T-kininogen. These molecules are synthesized by hepatocytes and released into plasma. They play a role in releasing kinin.

BK and kallidin or lysyl-BK are two peptides referred to as kinins. BK is a nonapeptide usually found in all secretions of the body, such as urine, saliva, and sweat. Also, it is found in several tissues, such as the heart, vasculature, blood, kidney, colon, and liver[2]. BK is produced by plasma kallikrein and can also be produced from kallidin by several aminopeptidases through cleavage of amino terminal lysine. On the other hand, kallidin is a decapeptide found in the heart, urine, and circulation[2]. Kallidin is produced by tissue kallikrein and is rapidly converted to BK by the enzyme aminopeptidase N.

In addition to BK and kallidin, there are two other kinin fragments: des-arg-BK or BK-(1-8), and des-arg-KD or KD-(1-9). They can interact with kinin receptors and function as agonists for type 1 BK receptors. They are stimulators of type 1 BK receptors. Moreover, BK-(1-7) is an inactive degradation product, whereas BK-(1-5) is involved in the coagulation system.

The purpose of this project is to discuss formation and pathophysiological roles of the kinin system, define the proinflammatory and the cardioprotective effects of the kinin system, outline the involvement of the kinin system in the regulation of the renin system, discuss the future prospects of kinin-related therapy for pathological conditions, and describe the undesired effects of kinins.

### FORMATION OF BRADYKININ

BK is a vasoactive peptide that is formed during inflammatory response from either HMW or LMW kininogen by action of enzymes called kallikreins. Three proteins are involved in BK formation: Hageman factor, prekallikrein, and HMW kininogen. Plasma prekallikrein circulates in a complex form with HMW kininogen. This complex, together with the Hageman factor, binds to negatively charged surfaces, including basement membrane components and proteoglycans such as heparin. Once they are exposed by tissue damage, prekallikrein is rapidly converted to plasma kallikrein by the enzyme prolylcarboxypeptidase. Kallikreins are the enzymes that break down kininogen (the precursors of kinin). There are two forms of kallikreins: plasma kallikrein, which converts HMW kininogen to BK, and tissue kallikrein, which converts LMW kininogen to lysyl-BK (kallidin), which is rapidly converted to BK by the enzyme aminopeptidase N as shown in Fig. 1.

### DEGRADATION OF BRADYKININ

Degradation of the BK peptides is done by enzymes called kininases. Kininases cleave BK at either their amino- or carboxyterminal end. Enzymes that cleave at aminoterminal are aminopeptidase M (APM), which can degrade kallidin into BK[3], and aminopeptidase P (APP), which cleaves the first amino acid of BK to give BK-(2-9).

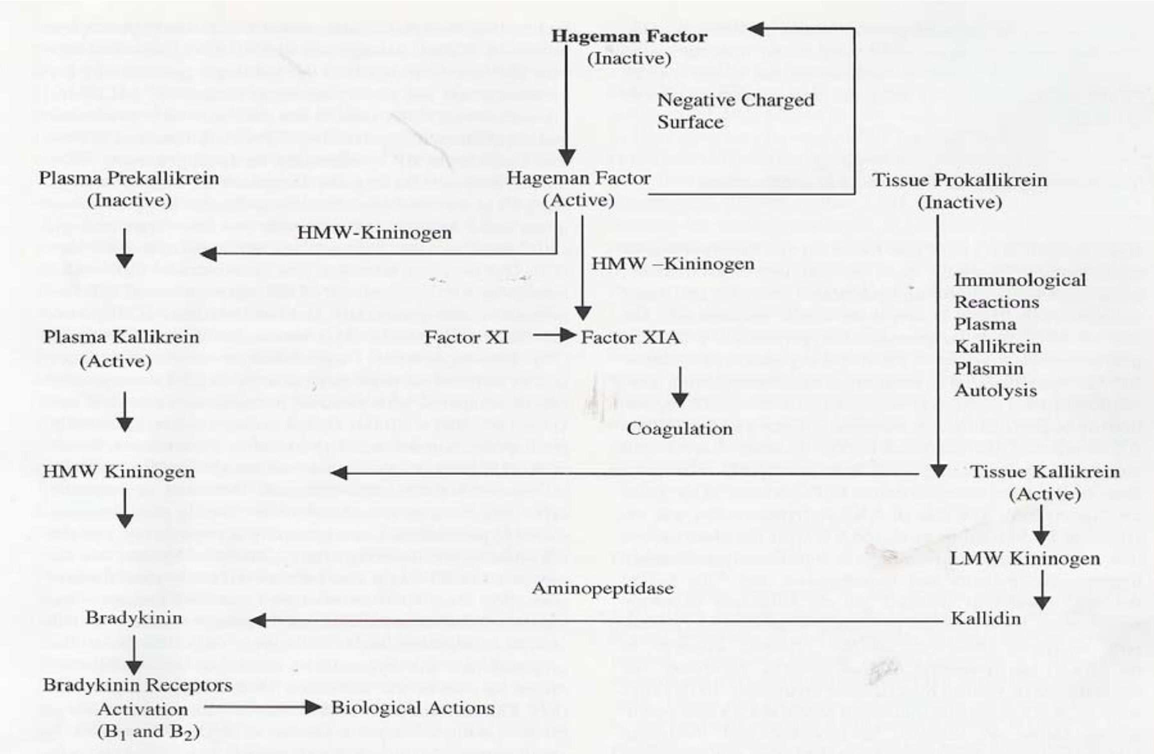

**FIGURE 1**. Mode of the kinin formation.

On the other hand, there are four major enzymes responsible for carboxyterminal degradation of BK: angiotensin-converting enzyme (ACE), carboxypeptidase N and M (CPN, CPM), and neutral endopeptidase (NEP). These kininases can be divided into two groups on the basis of their enzymology. The first group is kininase I, which includes CPN and CPM, and the second group is kininase II, which includes ACE and NEP. Enzymes of kininase I cleave the carboxyterminal arginine from either BK or kallidin to give des-Arg9-BK or des-Arg10-KD, while enzymes of kininase II cleave the dipeptide Phe8-Arg9 from both BK and kallidin to give BK-(1-7) and KD-(1-8). These are further cleaved by ACE to give BK-(1-5) and KD-(1-6).

### RECEPTORS FOR BRADYKININ

There are two subtypes of BK receptors, $B_1$ and $B_2$, according to IUPHAR classification. These two receptors have similar structures with seven transmembrane domains coupled to G-protein. Also, $B_3$ and $B_4$ receptors have been proposed as additional receptors[1,2].

$B_1$ and $B_2$ receptors are structurally similar, however, there are many differences in their functions in living organisms. To begin with, $B_1$ receptors are expressed at a very low level in healthy tissue, and they are inducible following tissue injuries and by endogenous factors (endotoxins, cytokines, growth factors), while $B_2$ receptors are predominant and constitutively expressed in vascular, nonvascular smooth muscles and the heart. In addition, $B_1$ receptors have no desensitization and internalization[4], whereas $B_2$ receptor desensitization is due to negative cooperativity, which is a phenomenon observed with G-protein–coupled receptors. Both receptors have been cloned and their structures elucidated. Moreover, signal transduction mechanisms are quite similar for $B_1$ and $B_2$ receptors, since they are coupled to G proteins so they require calcium for signaling[5].

### Physiological Effects of $B_1$ Receptors

BK $B_1$ receptors have roles in different systems. First of all, in the circulation, $B_1$ receptor stimulation can cause vasodilatation in the vessels. Also, in the cardiovascular system, $B_1$ receptors have been shown to precondition the heart against ischemic events and protect the heart from arrhythmias. In addition, $B_1$ receptors are involved in renal functions by affecting both natriuresis and glomerular filtration. Also, they are involved in the pathogenesis of diabetes. In inflammation, $B_1$ receptors are involved in leucocyte recruitment and in the initiation of inflammatory responses as well as in the physiology of pain. Finally, it has been shown that $B_1$ receptors are mitogenic in fibrotic tissues[6,7].

### Physiological Effects of $B_2$ Receptors

$B_2$ receptors produce different effects on a number of tissues. In vasculature, $B_2$ receptor signaling can lead to vasoconstriction or vasodilatation, and can cause either stimulation or inhibition of growth in parenchymal tissues. In the cardiovascular system, $B_2$ receptors have been shown to be antiarrhythmic in the heart and antithrombotic in the vasculature. Also, $B_2$ receptors reduce infarct size and they precondition the heart against ischemic events. Moreover, these receptors improve the myocardial demand of oxygen in heart failure by attenuating the endothelial dysfunction.

In addition, $B_2$ receptors affect other systems. In diabetes, they affect glucose metabolism either directly or by interaction with insulin. Also, BK and its receptors have a role in the alimentary tract. They affect smooth muscle cells of the duodenum, ileum, and cecum, causing either relaxation or contraction. Moreover, $B_2$ receptors have a role in the pathogenesis of asthma as they cause chloride secretion and bronchoconstriction. $B_2$ receptors also affect the functions of reproductive organs and the bladder by inducing smooth muscle contraction in the vas deferens, uterus, and bladder. Finally, $B_2$ receptors can be involved in the physiology and pathophysiology of pain, inflammation, and hyperalgesia.

## PATHOPHYSIOLOGICAL ROLE OF BRADYKININ

### Pain and Neurology

The pain-producing effect of BK is due to stimulation of afferent nerve terminals. This is caused by the presence of $B_2$ receptors on neural elements as in nonmylinated nerve terminals, sensory ganglions, and dorsal layer of the spinal cord; thus BK is both algesic and hyperalgesic[8]. Although these effects are mediated mainly by $B_2$ receptors, $B_1$ receptors are also involved in the process of pain perception since the $B_1$ receptor agonist [des-Arg9-BK] exacerbates the pain. BK-induced algesia and hyperalgesia have been shown using a number of models, such as BK-evoked pain in human blister base and in the BK-elicited vascular pain and cutaneus hyperalgesia in rat. Moreover, BK has been reported to cause depolarization in nerve fiber by the activation of sodium channels, and it is also involved in the chemokine-induced hyperalgesia by activation of both $B_1$ and $B_2$ receptors. In addition, pain related to local inflammation of the small intestine or bladder is mediated by endogenous BK through a viscero-visceral hyper-reflexia. This hyper-reflexia can be inhibited by the BK receptor antagonist. Also, expression of the $B_1$ receptor has been shown in inflammatory bowel diseases, such as ulcerative colitis and Crohn’s disease[9].

### Allergy

BK is involved in the pathogenesis of allergic asthma and bronchitis. Earlier reports indicated that injecting skin with BK may cause Lewis’s triple response, which is similar to that caused by histamine.

BK either released with the airway tissue or derived from the blood stream can stimulate sensory nerves to release tachykinin. Tachykinins cause bronchoconstriction and plasma exudation during the anaphylactic response. This involvement of tachykinin in BK-induced plasma leakage in the airway constitutes a neurogenic aspect of asthma[10].

#### Rhinitis

There is a lot of evidence showing that BK is involved in the symptoms of different types of rhinitis. Specific binding to $B_2$ receptors are found in the nasal turbinate of guinea pigs and man. Also by using a sensitive lavage method, elevated BK levels have been detected in the nasal cavity of man. The receptors involved in these effects are mainly $B_2$ receptors, since the selective $B_1$ receptor agonist (des-Arg9-BK) does not produce such effects. Moreover, the effects of BK on nasal cavity and lower airway are mediated partly by platelet activating factor (PAF).

#### Fever

Fever is a reaction to sepsis and inflammation in which BK and prostaglandins (PGs) may play a role. Intravenous administration of BK causes a febrile reaction in the rat and it is inhibited by i.c.v. injection of HOE 140, which is a $B_2$ receptor antagonist, and also by [Leu$^8$]-des-Arg9-BK, which is a $B_1$ receptor antagonist[10]. These data reported that endogenous BK in the central nervous system (CNS) has a role in thermoregulation process.

### GASTROINTESTINAL DISEASES

BK receptors are involved in many gastrointestinal (GI) diseases. First, the dumping syndrome that results as a complication of gastric surgery is characterized by flushing and hypotension, with excessive release of BK and tryptophan 5-hydroxylase from the gut. Moreover, the carcinoid syndrome results in excessive production of kallikrein and tryptophan 5-hydroxylase in enterochromaffin cells caused by intestinal tumors and hepatic metastases.

In addition, the release of PGs by BK increases the secretion of chloride and water that link BK to diarrhea in gastroenteritis. Also, PGs released by BK have a role in human adenocarcinoma. Both $B_1$ and $B_2$ receptors are involved in this pathological process. BK is also involved in the chronic inflammatory colonic mucosa and ulcerative colitis[9].

### PROINFLAMMATORY EFFECTS OF BRADYKININ

BK may play an important role in the development of the inflammatory process. It has been reported that this peptide caused signs of inflammation (redness, local heat, swelling, and pain) when injected into animal tissues. This inflammatory response to BK seems to be due to $B_2$ receptor activation, which causes vasodilatation due to production of NO and PGI, and also increased permeability of vascular endothelium to fluid and plasma proteins that result in edema. It is well known that $B_2$ receptors are mainly responsible for the development of signs of inflammation, thus $B_2$ receptor antagonists prevent specific inflammatory responses in specific models. Examples are tissue swelling and systemic signs of inflammation in peptidoglycan-induced arthritis in the Lewis rat and plasma extravasations in the Arthus reaction[11,12]. Also, $B_2$ receptor antagonists play a role in some forms of vasogenic edema, such as brain edema following head trauma or stroke, and hereditary angiodema. On the other hand, there is experimental evidence for the involvement of the up-regulation of $B_1$ receptors in chronic inflammatory processes, including periodontitis, rheumatoid arthritis, osteomyelitis, chronic cystitis, and $B_1$ receptors of BK are also involved in the neurogenic inflammation process. The reports have shown that tachykinin-mediated, capsaicin-evoked edema in the mouse ear is inhibited by the antagonist of $B_1$ receptor more than $B_2$ receptor. This suggests the existence of $B_1$ receptors on neurokinin nerves that modulate the neurogenic inflammation in these species. In addition, BK is responsible for many effects in leukocytes, including the release of other inflammatory mediators like cytokines, PGs, leukotriens, and reactive oxygen species. Also, an up-regulation of BK receptors on neutrophils and macrophages appears to be involved in increasing the sensitivity of these cells to BK at the site of inflammation.

Moreover, elevated BK levels are detected in the plasma of asthmatics. Increasing levels of BK have been found in symptomatic asthmatics subjects, but not in asymptomatic subjects. BK has direct and indirect airway actions that include neurogenic inflammation by stimulation of sensory nerve fibers, arteriolar dilatation, vasoconstriction, and plasma extravasations leading to mucosal edema, contraction of smooth muscles contributing to bronchoconstriction, and release of inflammatory mediators such as cytokine[10,13]. In addition, BK acting through $B_1$ and $B_2$ receptors may stimulate proliferation of a number of normal and neoplastic cell types through increasing oxygenation and nutrition of the proliferating tissue, thereby facilitating cell growth as in A431 carcinoma cells and small cell lung carcinoma cells. Also, BK may have mitogenic roles in a range of clinical conditions associated with cellular proliferation, including (1) psoriasis, which is characterized by excessive keratinocyte proliferation that produces hyperplasic skin plaque; (2) in the kidney, BK may contribute to the mesangial cell proliferation that may lead to glomerulonephritis; and (3) BK can stimulate vascular smooth muscle proliferation and it may contribute to the medial and intimal hyperplasia of hypertension and atheroschelorosis[13]. Similarly, the mitogenic action of BK may be involved in endometrial proliferation during the menstrual cycle and in lymphocyte proliferation, in addition to the enhancement of the immune response, which demonstrates the role of BK in multiple sclerosis.

### CARDIOPROTECTIVE EFFECTS OF BRADYKININ

BK has multiple effects on the cardiovascular system. These effects are produced through vasodilatation and plasma exravasation properties, which lead to inflammation[14]. Vasodilatation is mainly mediated by the $B_2$ receptor, however under inflammatory conditions, $B_1$ receptor up-regulation mediates BK-induced vasodilatation and hypotension. BK is a potent vasodilator; it acts through stimulation of endothelial cells, causing release of secondary mediators that affect the vascular smooth muscle. These mediators are NO and prostaglandin $I_2$ ($PGI_2$). NO is derived from L-arginine by endothelial NO synthase and it diffuses from endothelium to smooth muscle where it activates guanylate cyclase. $PGI_2$ (prostacyclin) formation occurs in many cell types through a cytosolic $Ca^{2+}$-sensitive isoform of phospholipase $A_2$, and it stimulates cyclic AMP production in the smooth muscle cell. Also, other mechanisms of endothelium-dependent vasorelaxation are suspected, such as the activation of a number of NO-independent ion channels located in the smooth muscle cells[15]. These physiological effects of BK are useful in the treatment of hypertension and ischemic disorders, in addition to maintaining renal function. Many studies were done on knockout mice that lack $B_2$ receptors and laboratories investigated the enhancement effect of endogenous, exogenous arginine vasopressin and exogenous angiotensin II[16]; distorted renal development in the fetus with abnormal distal nephrons, reduced glomerular capillary surface area, decreased rennin and cyclooxygenase COX 2 expression in the kidney[17]; insulin-resistance state and increased renal fibrosis in response to unilateral urethral obstruction[18]. All these findings support the protective role of the KKS in cardiovascular and renal disorders.

In addition, BK elevates the cardiac output and causes coronary dilatation, which is beneficial during myocardial infarction and the postinfarction period. The coronary dilatation induced by BK is mediated by the activation of $B_2$ receptors, since the $B_2$ receptor antagonist HOE140 inhibits the effects on the coronary arteries. Also, both NO and $PGI_2$ are involved in BK-induced coronary dilatation; in contrast, the $B_1$ receptor agonist (des-Arg9-BK) does not affect coronary blood flow.

Moreover, $B_2$ receptors are involved in the regulatory effect of NO on myocardial oxygen consumption. Recently, experimental evidence has suggested that G-protein–mediated "cross-talk" mechanisms between $B_2$ receptors, NO synthase enzyme, and angiotensin AT1 receptors may play an important role in cardioprotective effects of BK. Also, another interesting implication of BK is the ischemic preconditioning that is believed to strengthen their cardioprotective properties. It has been shown that $B_2$ receptor activation is involved in the experimental ischemic preconditioning in rabbit, guinea pig, and rat hearts. Moreover, BK exhibited pleiotropic effects by inhibiting apoptosis, inflammation, hypertrophy, and fibrosis, and promoting angiogenesis in the heart.

### INVOLVEMENT OF THE KININ SYSTEM IN THE REGULATION OF RENIN SYSTEM

The KKS and the rennin-angiotensin system (RAS) have many interactions as shown in Fig. 2.

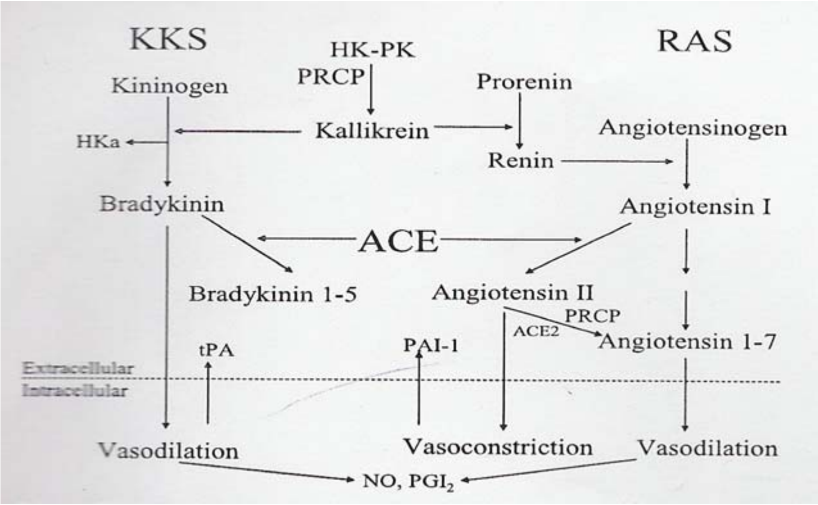

**FIGURE 2.** Interrelationships between bradykinin and angiotensin systems.

The first important link recognized between these two systems was ACE. This enzyme has the bifunctional activity of being one of the degrading peptidases (kininase II) of BK and converting the inactive 10-amino acid angiotensin I to the biologically active 8-amino acid angiotensin II, which induces local vasoconstriction and increases blood pressure. Recently, a homologue of ACE has been recognized, which is ACE 2[19]. ACE 2 is a carboxypeptidase located mainly in the heart, kidney, and testis. It degrades angiotensin I to angiotensin (1-9) by removing the COOH-terminal lysine. Angiotensin (1-9) enhances arachidonic acid release by BK and resensitizes the BK $B_2$ receptors. Moreover, the effects of ACE on BK metabolism have been shown by the influence of ACE inhibitors (ACE I) on a number of biologic processes. ACE I result in 25% reduction in death from cardiovascular disease, 20% reduction in myocardial infarction, 30% reduction of stroke, 22% reduction in heart failure, and 16% reduction in complications of diabetes[20]. Furthermore, BK elevation after ACE I treatment improves left ventricular diastolic dysfunction, improves insulin resistance, and reduces the progression of different fibrotic renal diseases in animal models.

In addition, it has been shown that there are interactions between angiotensin (1-7) and BK. Angiotensin (1-7) is produced by ACE 2 or by degradation of angiotensin II through prolylcarboxypeptidase. Also, neprilysin and thimet oligopeptidase can produce angiotensin (1-7) from the breakdown of angiotensin I. All these enzymes are involved in the BK metabolism. There are interactions between angiotensin (1-7) and the

KKS that have been studied in the kidney: potentiation of BK by angiotensin (1-7), mediation of vascular activity of angiotensin (1-7) by BK, potentiation of the hypotensive and vasodilatory effects of BK in normotensive or hypertensive rat, and dilating coronary artery through BK and NO. Moreover, angiotensin (1-7) influences BK by inhibiting ACE, stimulating BK $B_2$ receptors, and stimulating its own receptors that may interact with BK receptors.

Furthermore, interactions between plasma and tissue kallikrein with the RAS have been reported. Both plasma and tissue kallikrein are activators of prorenin. Plasma kallikrein is an essential enzyme for prorenin activation after factor XII activation of plasma. This activation can occur at neutral pH. However, tissue kallikrein has been reported as a prorenin-converting enzyme at pH 8.2. Also, mouse tissue kallikrein mk1, mk9, mk13, and mk22 were shown to be prorenin activators in addition to human tissue kallikrein hk1[21].

Recent studies indicate that the enzyme prolylcarboxypeptidase, which is an aniotensin II–inactivating enzyme, is also a prekallikrein activator. The ability of prolylcarboxypeptidase to act in the KKS and RAS indicates a novel interaction between them. Prolylcarboxypeptidase is a serine protease enzyme that is the first endothelial cell prekallikrein activator that has been identified. Its actions are formation of angiotensin (1-7), BK release, vasodilatation, and lowering blood pressure.

Furthermore, angiotensin II receptors have been implicated in the cross-talk between the RAS and the KKS. The finding first recognizes this evidence that the RAS stimulates renal BK production and cGMP formation through angiotensin II receptors 2 ($AT_2$). This shows that stimulation of $AT_2$ receptors results in releasing BK and NO. The ability of angiotensin II to directly stimulate renal BK production was shown in conscious rats and this effect is blocked by $AT_2$ receptor antagonists, not by $AT_1$ receptor antagonists. Moreover, in rat, $AT_2$ receptor stimulation induced a systemic vasodilator response mediated by BK and NO, which counterbalances the vasoconstrictor action of angiotensin II at the $AT_1$ receptor. This suggests that $AT_1$ receptor blockage potentiates the cardiovascular effects of ACE I in the heart and kidney[22].

Another potential interaction between the RAS and the KKS may be the modulation of the RAS by BK peptide level. It has been shown that BK administration increases renin secretion. This effect is mediated by increased NO formation. Also, it is reported that the administration of icatibant decreases the plasma renin level in anesthetized rabbits.

### FUTURE PROSPECTS OF BK-RELATED THERAPY FOR PATHOLOGICAL CONDITIONS

Over the past 2 decades, it has become clear that BK and their receptors are involved in several pathological conditions. As such, the development of $B_1$ and $B_2$ receptor antagonists will be available in the near future in order to provide more selective therapeutic modalities for the treatment of many diseases.

#### Agonists and Antagonists of $B_1$ Receptor

Since 1970, many studies were done to develop receptor antagonists. These studies are based on modifying BK sequences. $B_1$ receptor antagonists were discovered 10 years before $B_2$ receptor antagonists. The first family of the compounds responsible for antagonizing BK and des-Arg9-Bk was based on the prototype [Leu-8]-des-Arg9-BK; it also has specificity for $B_1$ receptor.

**TABLE 1**
**$B_1$ Receptor Agonists and Antagonists**

| Ligands | Application | Studies |
|---|---|---|
| **Agonists** | | |
| R-838(Sar-[D-Phe$^8$]des-Arg$^9$-BK) | Metabolically stable<br>High affinity and selectivity<br>Hypertension<br>Stimulation of vascular formation following ischemia | Rabbit<br>Rodent |
| **Antagonists** | | |
| [Leu$^8$]-des-Arg$^9$-BK | Pain<br>Ischemic vascular disease | Rat<br>Mice |
| Lys-[Leu$^8$]des-Arg$^9$-Bk | Optimal $B_1$ receptor antagonist | Human $B_1$ receptor antagonist |
| Ac-Lys-[MeAla$^6$,Leu$^8$]des-Arg$^9$-BK | Metabolically stable (not very potent compared with the affinity of reference compound Lys-[Lue$^8$]des-Arg$^9$-BK) | Rabbit |
| R-715(Ac-Lys-[βD-Nal$^7$,Ile$^8$]des-Arg$^9$-BK | High affinity<br>Allergic lung inflammation | Human and rabbit $B_1$ receptor<br>Mice |
| B9858(Lys-Lys-[Hyp$^3$,Igl$^5$,D-Igl$^7$,Oic$^8$]des-Arg$^9$-BK) | Fairly high selectivity for $B_1$ receptor due to Lys<br>Metabolically stable residue | Human and rabbit $B_1$ receptor |
| Des-Arg$^{10}$-HOE 140 | Residual antagonistic effects on B2 receptor<br>Moderate affinity | Rabbit jagular vein, guinea pig ileum, rabbit aorta |
| B9430 (D-Arg-[Hyp$^3$,Igl$^5$,D-Igl$^7$,Oic$^8$]-BK) | Mixed $B_1$ and $B_2$ receptor antagonist even if des-Arg$^9$ fragment has substantial selectivity for $B_1$ receptor | Demonstration of compatibility of $B_1$ and $B_2$ receptor structure by the accommodation of a single pharmacophore |
| R-954(Ac-Orn-[Oic$^2$,α-MePhe$^5$,D-βNal$^7$,Ile$^8$]des-Arg$^9$-BK) | Allergic lung inflammation<br>Airway allergy | Mice<br>Rat model |
| PS020990 | Potent and competitive $B_1$ receptor antagonist<br>High affinity | Human receptor (no *in vivo* data) |
| Compound 12 (benzodiazepine-based structure) | Selective antagonist | Human and rat $B_1$ receptor *in vitro*<br>Rat and dog |
| Benzo-sulfonylamide compounds<br>Compound 12<br>Compound 11<br>SSR240612 | Powerful and selective antagonist<br>Hyperalgesia<br>Speculative on pain, inflammation and sepsis<br>Inflammation and hyperalgesia | Rabbit aortic preparation, rabbit jugular vein<br>Mice and rat |

### Agonists and Antagonists of $B_2$ Receptor

The $B_2$ receptor was well defined when the first generation of antagonists were produced in 1985[23]. These compounds are based on [D-Phe$^7$]-BK and have agonist/partial antagonist activity with low potency.

The second generation $B_2$ receptor antagonists have high antagonistic activity compared to the first generation, in which rigidity had been added to the C-terminal region of the peptide by introducing non-natural amino acid residue, which gives them critical antagonism. In addition, the development of the third generation $B_2$ receptor antagonists has evolved toward nonpeptide drug development programs with oral bioavailability, higher lipophilicity, and lower molecular weight. Moreover, some $B_2$ receptor antagonists have been discovered as natural compounds, such as martinelline, which is a pyrroloquinoline alkaloid isolated from the plant *Martinella iquitosensis*[24].

Table 2 shows types of B2 receptors agonists and antagonists with their pharmacological and clinical application[30].

**TABLE 2**
**$B_2$ Receptor Agonists and Antagonists**

| Ligands | Application | Studies |
|---|---|---|
| **Agonists** | | |
| Labradimil ([Hyp$^3$, Thi$^5$, 4-MeTyr$^8$ Ψ(CH2-NH)Arg$^9$]-BK) | Vascular permeability (blood brain barrier): adjuvant to chemotherapy of brain tumors | *In vivo* rodent models<br>Human: phase II studies on glioma |
| FR190997 | Hypertension | Rat |
| **Antagonists** | | |
| First generation: | | |
| [D-Phe$^7$]-BK | Low potency, antagonist/ partial agonist activity | Rat uterus, guinea pig ileum |
| [Thi$^{5,8}$, D-Phe$^7$]-BK | Potent antagonist, no agonist activity | Rat uterus, guinea pig ileum |
| Second generation: | | |
| HOE 140 (Icatibant; D-Arg-[Hyp$^3$, Thi$^5$, D-Tic$^7$, Oic$^8$]-BK) | High affinity, long lasting, competitive activity<br>No residual agonist effect<br>Resistance to peptidases | Animal model (high affinity for the human, rabbit, and guinea pig $B_2$ receptor) |
| | Acute rhinitis | |
| | Asthma | Human, nasal treatment |
| | Early stage of inflammation | Human |
| | Persistent inflammatory pain | Rat |
| Third generation: | | |
| Phosphonium family: | | |
| WIN64338 | Inactive | On human tissue |
| | Limited affinity | For guinea pig $B_2$ receptor |
| WIN62318 | Micromolar binding affinity to human $B_2$ receptor | Identification of the absolute requirement for $B_2$ receptor binding affinity: presence of two positive charges at a distance about 10 Angstrom separated by a lipophilic residue, playing the role of the Phe$^8$ side chain in the native ligand |
| Quinoline and imidazole [1,2-α]pyridine family: | High $B_2$ receptor affinity and selectivity vs. $B_1$ receptor | Oral activity at doses ranging between 1 and 30 mg/kg in different tests and species |
| FR165649,<br>FR173657,<br>FR184280 | Oral activity on hyperalgesia and inflammation | Rat and mice |
| FR167344 | Selective and high potent binding activity | Guinea pig ileum, human A-431 cells |
| | Bronchoconstriction | Guinea pigs (oral activity) designed as clinical candidate to treat inflammatory disease |
| Compound 38 | High affinity | Human $B_2$ receptor |

**TABLE 2 continued**

| | | |
|---|---|---|
| CP2522 | High affinity<br>Modeled on CP0597 by replacing β-turn conformation of the peptide by a rigid 1,4 piperazine ring | Human $B_2$ receptor |
| Substituted 1,4-dihydropyridines | $B_2$ receptor antagonist at the nanomolar range | Human $B_2$ receptor |
| Bradyzide | Hypertension, inflammation | Rodent |
| Natural compound<br>Pyrroloquinoline alkaloid: Martinelline | Affinity for both $B_1$ and $B_2$ receptors at the micromolar range, but not selective | Alkakoid isolated from the South American tropical plant *Martinella iquitosensis* |
| L-755807 | Inhibition of bradykinin binding to cloned human $B_2$ receptor at micromolar range | Complex metabolite isolated from a culture of the mould *Microsphaeropsis* sp.<br>No future pharmacological data |

### BRADYKININ AND HUMAN DISEASES

#### Neurological Disease

BK has a significant role in a variety of CNS diseases, as $B_2$ receptors are widespread within the CNS vasculature. This characteristic leads to increased permeability and delivery of chemotherapeutic agents across the blood brain barrier[25]. Also, BK receptors play an important role in the regulation of post-traumatic cerebral edema, as it can increase blood brain barrier permeability. There are many studies done to show the effect of using $B_2$ receptor antagonists in the treatment of human traumatic brain injury. The first study was done by using the $B_2$ receptor antagonist CP-0127, which was infused in patients with severe focal cerebral contusions presenting 24 to 96 h after injury for 7 days[26]. Also, it has been reported that the mean peak increase in intracranial pressure, as well as mean increase in severity scores, was significantly less in treated patients. Moreover, BK receptors have been linked to Alzheimer's disease. The study shows that skin fibroblast derived from patients with familial Alzheimer's disease generated increased amounts of IP3 (inositol phosphate) in response to stimulation with BK.

#### Airway Disease

$B_2$ receptor is the major BK receptor involved in airway diseases. It has been reported that inhalation of $B_2$ receptor agonist BK or Lys-BK caused bronchospasms in asthmatic, but not in normal subjects, whereas weak $B_1$ receptor agonist des-Arg9-BK failed to cause bronchospasms in either asthmatic or normal subjects. In addition, $B_2$ receptor agonist of BK, but not des-Arg9-BK caused rhinitis symptoms. The role of $B_2$ receptors in asthma was shown by clinical trials of nebulized icatibant in patients with persistent asthma. It has been demonstrated that after 1 month of therapy, patients given a higher dose of icatibant had a mean increase of 10% in forced expiratory volume, which suggest an anti-inflammatory role of $B_2$ receptor antagonist, but no effect on bronchodilation was seen. Also, it had been demonstrated that using intranasal insufflation prior to nasal allergen challenge had no effect on the immediate allergic nasal response, but it can block histamine hyper-responsiveness at 24 h as well as allergen-induced increases in eosinophils, eosinophil cationic protein, kinin, and interleukin-8.

On the other hand, the potential role of the $B_1$ receptor in airway disease has been difficult to establish. It has been shown that the potent $B_1$ receptor agonist lys-des-Arg9-BK had no acute effect on either the upper or lower airway; however, in rat model of asthma, the $B_1$ receptor antagonist was found to inhibit allergen-induced bronchial hyper-responsiveness

This suggests that $B_1$ receptors may be involved in allergic airway inflammation. Also, a recent human study reported the expression of $B_1$ receptors in nasal tissue in allergic rhinitis subjects, but not in normal[27]. Also, expression of immunoreactive $B_1$ receptor was found from patients with interstitial lung disease in transbronchial biopsies, but not in normal lung tissue. In addition, patients taking ACE I experienced cough as a side effect. BK has a role in causing cough by acting through stimulation of PG, which is a possible mediator of ACE I–induced cough.

### Cancer

The role of BK receptors in human cancer has been studied, as BK can stimulate growth and increase vascular permeability of tumors. Increased generation of BK has been reported in several types of cancers. Cervical cancer tissue as well as cervical cancer metastatic lesions showed higher expression of both $B_1$ and $B_2$ receptors than normal cervical tissues, in contrast, prostate cancer tissue has been found to express high levels of $B_1$ receptor compared with normal prostate tissue, as $B_1$ receptors promote cell growth and stimulate migration as well as invasion in a prostate cell line[28].

Moreover, normal brain tissue expresses $B_2$ receptor on cortical neurons, but not on glial cells, unlike brain tissue with astrocytoma, which showed $B_2$ receptor on the astrocytic cells as well as variable expression of $B_1$ receptor.

In addition, BK receptor antagonists have been proposed for different cancers, especially lung and prostate cancers. The novel BK antagonist CU201 was shown to induce apoptosis and growth inhibition in various lung cancers and other cancer cells as well as its ability to inhibit the growth of prostate cancer in xenograft in nude mice.

In addition, studies showed that BK antagonists play an important role in cancer. B9870 is the most studied BK antagonist dimer as anticancer agent, at which two molecules of antagonist B9430 are joined at the N-terminus. This dimer is selectively cytotoxic at low concentrations for many types of cancer cells, whereas it does not damage normal cells. Other BK antagonists having anticancer activity are B-9870, B-10054, and M-516.

Finally, the discovery that BK antagonists can act synergistically with anticancer drugs opens an exciting new avenue for anticancer drug development. Also the fact that aspirin and other NSAIDs inhibit cancer growth may be directly related to recent results with BK antagonists since BK-evoked PG production[28].

### Hypertension

Hypertension is a major risk factor for the development of cardiovascular diseases like coronary heart disease, congestive heart failure, peripheral vascular, and renal diseases. It has been shown that the KKS exerts a fine control on vascular smooth muscle tone and arterial blood pressure, and plays a significant cardioprotective effect. Moreover, BK has a vasodilator action on peripheral blood vessels, and has potent diuretic and natriuretic effects that regulate sodium excretion from the kidney[14]. The deficiency of the KKS may participate in the genesis of hypertension. The involvement of BK in blood pressure regulation has been confirmed in transgenic mouse models with overexpression of human BK $B_2$ receptors. This overexpression of BK $B_2$ receptors caused the development of sustained lifetime hypotension. Also, B1 receptors may be involved in the blood pressure lowering effects under special situations.

It was reported that administration of aprotinin, a tissue kallikrein inhibitor or icatibant, a specific $B_2$ receptor antagonist, to the transgenic mouse restored blood pressure to normal levels. Also, kininase II (ACE) inhibitors like captopril and enalapril are currently used drugs in the treatment of hypertension[29]. Kininase II inhibitors lower blood pressure by blocking the conversion of angiotensin I

to angiotensin II and increasing the levels of BK[29]. Moreover, it has been demonstrated that $B_2$ receptor antagonist FR-173657 significantly abolished the hypotensive action of captopril; hence, these drugs should be contraindicated in patients with hypertension. Recently, it has been proposed that tissue kallikrein gene delivery into several hypertensive models results in reduction of blood pressure with the enhancement of capillary growth in spontaneously hypertensive rats, attenuation of cardiac hypertrophy, inhibition of renal damage, and fibrosis. These findings may indicate the prospects of kallikrein gene therapy for cardiovascular and renal pathology[29].

#### Hereditary Angioedema (HAE)

HAE is characterized by hereditary deficiency in $C_1$ inhibitor in contact and complement systems. It is associated with increased generation of $B_2$ receptors since the activity of kallikrein is largely dependent on the contact and complement systems[24]. The $B_2$ receptor antagonist icatibant is currently in trials for the treatment of attacks of angioedema in HAE patients. Also, aprotinin is a naturally occurring, 58 amino acid serpin. It acts by inhibiting the plasma kallikrein, which triggers the release of BK during the contact system activation leading to decreased release of BK during HAE attack. Another drug is DX88, which is a synthetic kallikrein inhibitor based on a recombinant, serine protease inhibitor domain. This drug reverses the increase in vascular permeability in $C_1$-inhibitor deficient mice.

#### Diabetes

Type 1 diabetes (insulin-dependent diabetes mellitus) is an inflammatory, autoimmune disease associated with vascular permeability changes leading to many complications. Recently, it was reported that BK $B_1$ receptors were found to be up-regulated in type 1 diabetes. This effect was recognized when type 1 diabetes was induced in male CD-1 mice by a single injection of streptozotocin, which showed expression of $B_1$ receptors in peripheral tissues with various functional consequences when exogenous des-Arg9-BK ($B_1$ receptor agonist) was administered. Interestingly, this type of diabetes in mice is associated with a state of hyperalgesia, which suggests some activation of the endogenous KKS. These effects were reversed by $B_1$ receptor antagonists like R-954 or R-715, which prevent the progression of insulin-dependent diabetes and also reversed hyperalgesia[23].

### UNDESIRED EFFECTS OF BRADYKININ

Many studies reported that BK has many undesired effects. First of all, BK showed pronounced proinflammatory properties *in vivo*[14]. These effects are due to activation of cyclooxygenase COX-1 or COX-2 and synergism with $PGI_2$. It has been reported that injecting BK into the skin of experimental animals and human volunteers mimics the cardinal signs of inflammation, which are redness, swelling, heat, and pain, as well as the accumulation of WBCs. Also, BK causes increased vascular permeability into the dorsal skin of rats on intradermal injection, increased paw swelling on subcutaneous injection into the hind paw of mice, writhing reactions recognized in mice on intraperitoneal injection, and increased angiogenesis as well as granulation on topical application in mice sponge implants. Other studies have suggested the role of BK and des-Arg9-BK in chronic inflammation as in arthritis. Moreover, thermal and mechanical hyperalgesia have been recognized with BK $B_1$ receptors. Recently, it has been demonstrated that increasing concentration of BK enhances rolling and adhesiveness of neutrophils in the mesenteric capillaries of rats.

In addition, the use of ACE I is associated with various adverse effects, and some of these are associated with the procedures that cause contact activation of BK, as ACE I are the main BK-inactivating metallopeptidases in humans. Angioedema is a life-threatening side effect that is observed with ACE I therapy. The pathogenesis of ACE I–induced angioedema is related to BK and its active metabolite des-Arg9-BK. Also, cough that occurs in 5–20% of patients has been hypothetically attributed to accumulation of endogenous BK. Furthermore, an anaphylactoid or hypersensitivity reaction observed during hemodialysis is another acute side effect of ACE I. Studies have demonstrated an increase in BK concentration in the plasma of patients during acute hypersensitivity reactions. Table 1 shows types of $B_1$ receptor agonists and antagonists with their pharmacological and clinical applications[30].

### CONCLUSION

BK is an active endogenous peptide that is involved in a number of pathophysiological conditions, such as pain, fever, GI diseases, and rhinitis, and shows proinflammatory, in addition to cardioprotective, properties. It has been shown that BK acts through two receptors, $B_1$ and $B_2$, that differ in the mechanism by which they are regulated. The development and use of both $B_1$ and $B_2$ receptor antagonists as potential drug targets has been implicated in several pathophysiological conditions like hypertension, airway diseases, cancer, HAE, and diabetes. Recently, it has been reported that there are links between the KKS and RAS, which results from the interactions of their multiple components. Although BK has multiple beneficial effects, some undesirable effects have been documented, such as inflammatory responses, edema, pain, and angioedema, which can be over come by BK antagonists. Finally, a number of other applications are awaiting clear clinical conclusions about the applicability of BK receptor antagonists in inflammatory bowel diseases, asthma, allergy, brain edema, and sepsis.
